# Non-melanoma skin cancer and solar keratoses. I. Methods and descriptive results of the South Wales Skin Cancer Study.

**DOI:** 10.1038/bjc.1996.534

**Published:** 1996-10

**Authors:** I. Harvey, S. Frankel, R. Marks, D. Shalom, M. Nolan-Farrell

**Affiliations:** Department of Social Medicine, University of Bristol, UK.

## Abstract

This study aimed to describe the prevalence and incidence of solar keratoses and skin cancers and the natural history of solar keratoses in a random population sample. It was a cross-sectional study, with follow-up, conducted in South Wales, and involved 1034 subjects aged 60 years and over drawn from the Family Health Services Authority register. The main outcome measures were detection of the presence of solar keratoses and skin cancers on sun-exposed skin and photographic validation of solar keratoses and biopsy confirmation of cancers wherever possible. We found that solar keratosis prevalence was 23% (95% confidence interval 19.5-26.5) and that of skin cancer (all types) 2% (95% confidence interval 1.0-3.5). The incidence rate of solar keratoses was 149 lesions per 1000 person-years and of non-melanoma skin cancer 9 per 1000 person-years. In all 21% (95% CL 16-26) of solar keratoses regressed spontaneously during follow-up. None underwent malignant change. We believe that the failure of individuals to seek medical advice and the variable under-registration of non-melanoma skin cancer makes population-based study important. The high prevalence and incidence of malignant and pre-malignant skin lesions in this random sample raise major public health concerns. The high rate of spontaneous regression of solar keratoses and the low rate of malignant change challenges conventional views about the need for routine treatment of these lesions.


British Journal of Cancer (1996) 74, 1302–1307
© 1996 Stockton Press All rights reserved 0007–0920/96 $12.00

### Non-melanoma skin cancer and solar keratoses. I. Methods and descriptive results of the South Wales skin cancer study

I Harvey[1], S Frankel[1], R Marks[2], D Shalom[2] and M Nolan-Farrell[3]

[1]*Department of Social Medicine, University of Bristol, Canynge Hall, Whiteladies Road, Bristol BS8 2PR;* [2]*University of Wales College of Medicine, Heath Park, Cardiff CF4 4DH;* [3]*South Glamorgan Health Authority, Temple of Peace and Health, Cathays Park, Cardiff CF1 3XN, UK.*

**Summary** This study aimed to describe the prevalence and incidence of solar keratoses and skin cancers and the natural history of solar keratoses in a random population sample. It was a cross-sectional study, with follow-up, conducted in South Wales, and involved 1034 subjects aged 60 years and over drawn from the Family Health Services Authority register. The main outcome measures were detection of the presence of solar keratoses and skin cancers on sun-exposed skin and photographic validation of solar keratoses and biopsy confirmation of cancers wherever possible. We found that solar keratosis prevalence was 23% (95% confidence interval 19.5–26.5) and that of skin cancer (all types) 2% (95% confidence interval 1.0–3.5). The incidence rate of solar keratoses was 149 lesions per 1000 person–years and of non-melanoma skin cancer 9 per 1000 person–years. In all 21% (95% CL 16–26) of solar keratoses regressed spontaneously during follow-up. None underwent malignant change. We believe that the failure of individuals to seek medical advice and the variable under-registration of non-melanoma skin cancer makes population-based study important. The high prevalence and incidence of malignant and pre-malignant skin lesions in this random sample raise major public health concerns. The high rate of spontaneous regression of solar keratoses and the low rate of malignant change challenges conventional views about the need for routine treatment of these lesions.

**Keywords:** non-melanoma skin cancer; solar keratoses; epidemiology; public health; ultraviolet light

Skin cancer is currently the subject of detailed attention in Britain. Identified by the government's *Health of the Nation Strategy* on account of its rising incidence and apparently high potential for prevention (Anonymous, 1992; Anonymous 1993), additional concern has resulted from the anticipated health effects of the thinning of the ozone layer. The target of this strategy is to halt the year-on-year increase in incidence of skin cancer by the year 2005,

There is strong evidence, based upon mortality and cancer registration data that malignant melanoma (MM) has become more common in many countries over the last 30 years (Streetly and Markowe, 1995; MacKie *et al.*, 1985; Popescu *et al.*, 1990; Bonett *et al.*, 1989; Elder, 1995). However, trends in non-melanoma skin cancer (NMSC) are less certain, largely because many cancer registers do not include these tumours, and for those that do under-ascertainment is a significant problem (Beadle *et al.*, 1982). Two Australian studies not based on cancer registers do, however, indicate a recent increase in NMSC incidence (Giles *et al.*, 1988; Marks *et al.*, 1993).

Despite the intense interest in skin cancer in Britain, the basic descriptive and analytical epidemiology of NMSC and solar keratoses (SK) – the latter being strong risk markers for subsequent development of both NMSC and MM (Marks, 1995) – is very sparse (Harvey *et al.*, 1989). Incident cases of NMSC outnumber MM, which is more comprehensively researched, by a factor of at least 8 : 1 (Anonymous, 1993). There has been only one truly population-based study of NMSC and SK from Northern Europe published in recent years (O'Beirn *et al.*, 1968). All other studies have used subjects seeking medical advice. These are inherently less satisfactory, since the proportion of those with NMSC and SKs who seek help is uncertain and the propensity to seek medical advice may vary over time (Harvey *et al.*, 1989). Additionally, a number of hypothesised risk factors for NMSC, such as Celtic ethnic origin, remain largely uninvestigated (Lane-Brown *et al.*, 1971; Marks, 1986).

Correspondence: I Harvey
Received 11 December 1995; revised 7 May 1996; accepted 14 May 1996

Thinning of the ozone layer is occurring at 4% per decade over northern latitudes (Armstrong, 1994) and threatens to exacerbate the growing skin cancer problem. A 1% stratospheric ozone depletion should in theory lead to a 1.14% increase in ground level ultraviolet radiation (UVR) at mid latitudes, and a 1–3% increase in both non-melanoma and melanoma skin cancers (Dahlback and Moan, 1990). Pollution in the lower atmosphere may exert a partial compensatory effect on UVR levels, however (Bruhl and Crutzen, 1989).

In summary, there is a pressing need in the UK for epidemiological investigation of NMSC and SKs in random population samples in order to provide baseline data against which the effectiveness of currently advocated preventive measures may be judged, and to identify and confirm hypothesised epidemiological risk factors.

The fieldwork for such a longitudinal study was performed in South Glamorgan, in South Wales, between 1988 and 1992. The principal objectives of this study were:

(1) to describe the prevalence and incidence of SKs and skin cancers in a random population sample;
(2) to describe the natural history of SKs;
(3) to identify risk markers for prevalent SKs and SCCs, for incident SKs, and for spontaneous remission of SKs;
(4) to evaluate, using observational epidemiological methods, currently recommended preventive measures.

The first two objectives will be dealt with in this paper and the third and fourth in a companion paper (Harvey *et al.*, 1996).

### Methods

A random sample of 1034 subjects age 60 years and over living in the county of South Glamorgan (total population 403 000) was drawn from the South Glamorgan Family Health Services Authority (FHSA) register. Only a small proportion of the elderly are not registered with a general practitioner in the UK (Robert *et al.*, 1995). Research ethics committee approval was obtained.

A letter was sent to each subject's general practitioner asking if they had any objection to the inclusion of the patient in the study. A brief explanatory letter was then sent to each subject proposing a time when a research registrar in dermatology would make a home visit. A tear-off slip and reply-paid envelope were provided as well as a telephone contact number. The registrar attempted to visit all those who did not indicate unwillingness to participate. Several attempts were made to contact subjects, involving further house calls, letters and telephone calls.

At the visit a detailed administered questionnaire was completed (seeking information about hypothesised risk factors for NMSC and SKs) and an examination made of the skin of the head and neck, arms (to the shoulder), legs (below the knee) and feet. Polaroid photographs and 35 mm slides were taken of suspected solar keratoses and skin cancers. Given that over 80% of SKs (Frost and Green, 1994) and over 75% of NMSCs (Gallagher *et al.*, 1990; Chuang *et al.*, 1990) occur on sun-exposed areas of the skin, this limited skin examination gave a high probability of detecting lesions with minimum disruption and embarrassment for these elderly study subjects. The 35 mm slides were later used to validate the diagnoses made by the registrar, by projecting a random sample for a panel of three consultant dermatologists who gave diagnoses independently of each other. The GP of any patient with a suspected skin cancer was contacted directly. Patients with SKs alone were reassured that the lesion did not require immediate intervention but would be assessed at a second visit. They were, however, advised to consult their GP if lesions showed change.

Skin type was assessed by asking subjects how their skin reacted after first unprotected exposure to the sun in spring/early summer. Subjects were asked to recall their natural hair colour at age 15 years. Cumulative UV exposure was assessed by asking subjects to estimate their average outdoor exposure (for weekdays and weekend days separately) during early adult life (20–39 years), middle age (40–59 years) and old age (60 years+). From this a cumulative number of hours of UV exposure was calculated. This approach was based on that used in previous epidemiological studies of skin cancer (Graham *et al.*, 1985) and takes account of the substantial UV radiation exposure that can occur on cloudy days (Marks, 1990). Skin colour was assessed by examining the inner aspect of the upper arm, an area of skin which is unlikely to be tanned.

A second, follow-up visit was made between one and two years later. A second questionnaire was completed, including questions about treatment received for any skin conditions and lesions in the intervening period, and the same skin areas were examined. New lesions were noted. The polaroid photographs taken at the first visit were used as an aid in determining whether SKs were new, persistent or had resolved. Local pathology department records were scrutinised to determine the histological nature of any lesions removed during the follow-up period. The patient's GP was also contacted when pathology records failed to provide information.

Information about skin type, hair colour at age 15 years and estimated cumulative UV exposure was obtained on both visits in order to examine the test–retest reliability of subjects' responses.

Data were coded, entered and analysed using SPSS for Windows version 6.

### *Sample size*

The key measure used to determine the sample size was an estimated prevalence of solar keratoses. Assuming a prevalence of 10.6% (as found in a previous study in Ireland, O'Beirn *et al.*, 1968), and aiming to have 95% confidence limits around this point estimate no wider than ±2.5%, required 580 subjects. Allowing for 20% non-response and a further 20% for FHSA register inaccuracy (Bickler *et al.*, 1993), a random sample of 1034 subjects was drawn from the register.

### *Statistical methods*

Confidence intervals for proportions, prevalence and incidence rates were determined using Confidence Interval Analysis (CIA) version 1.1. Agreement for categorical data was quantified using crude percentage agreement and Cohen's kappa statistic (Altman, 1991). Cohen's kappa provides a measure of agreement beyond that expected due simply to chance.

Multiple logistic regression was used to determine factors which were significant ($P<0.05$) independent predictors of binary outcome variables (such as response/non-response).

### Results

### *Response rates*

Where there was positive evidence (a letter/telephone call, or the original letter returned marked 'not known') that the subject had moved away from the address or had died, subjects were deemed to be unavailable to the study. A total of 23.4% of subjects were not available at the outset of the study (visit 1). Of those available, 70.7% (560) were seen at visit 1. Of these 560 subjects, 12% became unavailable during the follow-up period. Of those available for round 2, 79.3% were seen. The mean (median) follow-up interval was 1.43 (1.40) years.

Data on address (including postcode), sex and age were known for all subjects. Univariable analysis shows that round 1 non-responders were significantly more likely to be female, older and living in the inner city, than responders. Analysis by multiple logistic regression shows that these associations remained after adjustment for each other. Adjusted odds ratios (ORs) for response were: females *vs* males OR=0.70; 80+ years *vs* 60–64 years OR=0.58; inner city residence *vs* other OR=0.45.

Non-responders in round 2 were more likely to be older, but there was no difference between the sexes, nor between those living in the inner city and suburbs, either before or after adjustment for age. Round 2 non-responders were also more likely than responders to have had SKs/NMSCs detected in round 1, an association that was not independent of age on multiple logistic regression analysis.

### *Slide validation of diagnoses*

The majority view of the three validating consultant dermatologists was taken as the 'gold standard'. Altogether, 160 randomly selected slides were shown. Crude agreement was 86.3% (138/160). A total of 135/154 SKs diagnosed by the researcher were confirmed as SKs by the panel [predictive value=87.7% (95% confidence interval 83–93%]. The validators diagnosed 137 SKs in total [89% (95% CI 84–94%) of the number diagnosed by the researcher].

### *Demographic characteristics of study subjects*

These are shown in Table I. The mean (median) age when first seen in the study was 71.2 (69.3) years.

### *Skin, pigmentation and UV exposure characteristics of subjects*

The main results are shown in Table II. Subjects were asked about their skin type, hair colour at age 15 and their cumulative lifetime exposure to outdoor conditions at both visits. On the second occasion the researcher was blind to the response given on the first. For skin type, crude agreement was 61% (Cohen's kappa=0.43). For UV exposure (divided into quintiles), crude agreement was 37%, and 79% of subjects lay within one quintile of their round 1 assessment (Cohen's kappa=0.21). For hair colour crude agreement was 63% (Cohen's kappa=0.52).

Some 43% (241/560, 95% CI 39–47%) of subjects either never tan or tan with difficulty. Mean (median) cumulative

UV exposure was 59 450 (54 450) h, equivalent to a mean of 2.3 h exposure per day during the lifetime of a study participant of mean age (71.2 years). A total of 3.8% (21/560, 95% CI 2.3–5.7%) of subjects reported sunburn during the previous 2 years and 4.8% (27/559, 95% CI 3.2–7.0%) were current users of sunbeds or UV lamps. Of the total, 34% (188/560, 95% CI 30–38%) claimed to use sunscreen regularly.

### *Descriptive epidemiology*

*Solar keratoses* The principal findings are shown in Table III. The crude prevalence of solar keratoses was 23% (129/560) at the first examination (95% CI 19.5–26.5%), with a median of two SKs per person (range 1–17). Of these 31% were on the head and neck and 44% on the forearms. Age/sex-specific prevalence of SKs is shown in Table IV. Some 12.6% of subjects (49/390, 95% CI 9.3–15.9%) developed at least one new SK during the follow-up period.

A total of 557 person–years of follow-up were available in the study. The incidence of new SKs during the follow-up period was 149 per 1000 person–years (95% CI 119–178). Considering the number of persons affected by new SKs (rather than the number of new lesions) the incidence was 88 newly affected individuals per 1000 person–years (95% CI 63–113).

Twenty-one per cent (50/239, 95% CI 16–26%) of SKs present at the first visit regressed spontaneously by the time of the second visit. A total of 334 solar keratosis–years of observation were available from the study (assuming that SK regression occurred on average halfway through the follow-up period), which provides the denominator for calculation of the following rates. The SK regression rate is 150 per 1000 SKs per year (95% CI 111–188). None underwent malignant change. This permits 95% confidence that the true rate of malignant transformation of SKs lies between 0 and 11 per 1000 SKs per year.

*Skin cancer* The prevalence of NMSC was 1.6% (9/560, 95% CI 0.74–3.0%) with a basal cell to squamous cell ratio of 8:1. Of these, 0.36% (2/560, 95% CI 0.043–1.29%) had malignant melanoma. Histological confirmation was available for 8/11 of the subjects with clinically diagnosed skin cancer in round 1. No lesions other than prevalent lesions identified during the first visit were removed by either dermatologists or GPs during the follow-up interval.

At the follow-up visit five subjects were clinically judged to have developed new skin cancers, in all cases NMSC (basal cell carcinomata). Three subjects subsequently attended for medical attention and all had their lesions histologically verified. The incidence rate of new NMSCs was therefore 8.98 per 1000 person–years (95% CI 2.9–20.8) and there were no new MMs (95% CI 0–6.6 per 1000 person–years).

**Table I** Demographic characteristics of study subjects

| *Variable* | *Categories* | *Number* | *Percentage* |
|---|---|---|---|
| Age (years) | 60–64 | 138 | 25 |
| | 65–69 | 159 | 28 |
| | 70–74 | 90 | 16 |
| | 75–79 | 92 | 16 |
| | 80+ | 81 | 15 |
| Sex | Male | 234 | 42 |
| | Female | 326 | 58 |
| Social class | I | 41 | 7.3 |
| | II | 125 | 22.4 |
| | III non-manual | 95 | 17.0 |
| | III manual | 170 | 30.4 |
| | IV | 87 | 15.6 |
| | V | 31 | 5.5 |
| | Unclassifiable | 10 | 1.7 |
| Marital status | Married | 324 | 58 |
| | Single | 35 | 6.3 |
| | Divorced/separated | 15 | 2.7 |
| | Widowed | 185 | 33.1 |

**Table II** Skin, pigmentation and ultraviolet exposure characteristics of subjects

| *Variable* | *Categories* | *Numbers* | *Percentages* |
|---|---|---|---|
| Skin pigmentation | Negro/Asian | 5 | 0.9 |
| | Dark | 23 | 4.1 |
| | Medium | 279 | 49.8 |
| | Fair | 253 | 45.2 |
| Skin type (reaction to sun) | Never tans, always burns | 46 | 8.2 |
| | Tans with difficulty burns easily | 195 | 34.9 |
| | Tans easily, burns rarely | 237 | 42.4 |
| | Always tans, never burns | 76 | 13.6 |
| | Genetically brown | 3 | 0.5 |
| | Genetically black | 2 | 0.4 |
| Eye colour | Blue/green | 386 | 69 |
| | Brown/grey | 174 | 31 |
| Cumulative sun exposure (h) | 0–29 999 | 32 | 5.7 |
| | 30 000–49 999 | 198 | 35.4 |
| | 50 000–69 999 | 185 | 33.0 |
| | 70 000–89 999 | 79 | 14.1 |
| | 90 000–109 999 | 38 | 6.8 |
| | 110 000+ | 28 | 5.0 |
| Sunburn during the last 2 years | Yes | 21 | 3.8 |
| | No | 539 | 96.2 |
| Current use of sunbeds/ sunlamps | Yes | 27 | 4.9 |
| | No | 532 | 95.1 |

### Discussion

Skin cancer, allowing for under-registration, is probably the most commonly occurring cancer in Britain (Harvey *et al.*, 1989) and its incidence is generally agreed to be rising. It is no exaggeration to think of this as an epidemic (an occurrence in excess of expectation), although this terminology is more usually found in Australian descriptions of the problem (Marks, 1987). Recognising the magnitude of the threat to the public health, the UK government health strategy, *The Health of the Nation*, has proposed the ambitious target of halting the increase in incidence in the UK of all types of skin cancer by the year 2005 (Anonymous, 1993). Target setting is the simplest part, however, of a more complex process. It is clear in the case of skin cancer that important issues remain unaddressed concerning both the data requirements to monitor progress towards this target and the effectiveness of many of the preventive measures that are at present being widely implemented (Melia *et al.*, 1994). The South Wales study reported here is intended to contribute to both these areas.

Certain methodological matters require comment. The age group chosen for this study and the areas of the body examined were based upon the known sharp increase in prevalence and incidence of SKs and NMSCs over age 60 and the known anatomical distribution of the lesions (Scotto and Fears, 1978). The proportion of subjects in the random sample who were not available owing to change of address or death (23.4%) is similar to that found by Bickler and Sutton (1993) in inner London (26.7%), but higher than that reported (Roberts *et al.*, 1995) from Nottingham (up to 8.7%). It is likely that accuracy of FHSA registers has improved significantly since our study was performed as a result of efforts to improve their validity. The response rates were broadly comparable with other studies undertaken in this age group (Marshall, 1987), but it is clear that the

**Table III** Prevalence and incidence rates of skin lesions

| *Type of lesion* | *Measure of frequency* | *Point estimate (95% confidence limits)* |
|---|---|---|
| Solar keratoses | Prevalence | 129/560 = 23% (19.5–26.5) |
| Basal cell carcinoma | Prevalence | 8/560 = 1.4% (0.6–2.8) |
| Squamous cell carcinoma | Prevalence | 1/560 = 0.2% (0.006–1.0) |
| Malignant melanoma | Prevalence | 2/560 = 0.4% (0.04–1.3) |
| Solar keratoses: new lesions | Incidence (lesion-based)[a] | 83 new SKs = 149/1000 person–years (119–178) |
| Solar keratoses: new lesions | Incidence (person-based)[b] | 49 subjects = 88/1000 person–years (63–113) |
| Solar keratoses: regression | Regression rate | 50 SKs regressed = 150/1000 SKs per year (111–188) |
| Solar Keratoses: malignant change | Transformation rate | Zero SKs transformed = 0/1000 SKs per year (0–11) |
| Basal cell carcinoma: new lesions | Incidence rate | 5 new lesions = 8.98/1000 person–years (2.9–20.8) |
| Squamous cell carcinoma: new lesions | Incidence rate | No new lesions = 0/1000 person–years (0–6.6) |
| Malignant melanoma: new lesions | Incidence rate | No new lesions = 0/1000 person–years (0–6.6) |

[a] Incidence based upon number of new lesions. [b] Incidence based upon number of subjects affected by new lesions.

**Table IV** Age/sex-specific prevalence of solar keratoses

| | *Age group (years)* | *Prevalence of SKs % (denominator)* |
|---|---|---|
| Male | 60–64 | 17 (58) |
| | 65–69 | 24 (72) |
| | 70–74 | 35 (37) |
| | 75–79 | 50 (42) |
| | 80+ | 64 (25)[a] |
| Female | 60–64 | 10 (80) |
| | 65–69 | 6 (87) |
| | 70–74 | 25 (53) |
| | 75–79 | 26 (50) |
| | 80+ | 23 (56)[b] |

[a]Chi-square test for linear trend: $P < 0.001$. [b]Chi-square test for linear trend: $P < 0.000001$.

tendency of older subjects not to participate is likely to bias the results in the direction of generally lower prevalence and incidence rate estimates. This is particularly so for the follow-up visit, where only 56% of the originally eligible subjects agreed to be examined.

Histological confirmation was available for the majority of NMSCs diagnosed in this study, as has been widely recommended (Green *et al.*, 1988; Nixon *et al.*, 1986). The proportion of subjects for whom histology was available (8/11) is similar to the reported proportion of subjects with suspicious lesions attending for definitive diagnosis as part of skin cancer screening programmes (Krol *et al.*, 1991). Australian evidence indicates that, in the case of SKs, a clinical diagnosis arrived at by experienced dermatologists is confirmed in 94% of cases by histology (Ponsford *et al.*, 1983), rendering histological confirmation of these lesions less important. The agreement in diagnosis of SKs between the research registrar and the panel of validating dermatologists in this study (86%) was satisfactory. One limitation, however, is that the photographic validation only allowed assessment of the positive predictive value (the proportion diagnosed as SK actually having SK) of a diagnosis of SK. The negative predictive value (the proportion diagnosed as not having SK who truly do not), which should ideally be 100%, cannot be assessed from the data collected.

Many of the key exposure variables in this study consisted of self-reported data of a type which are inherently difficult to validate. Nonetheless, there are good grounds for using this approach. Cumulative sun exposure was assessed using methods used in several previous studies (Vitasa *et al.*, 1990; Gafa *et al.*, 1991; Urbach and Vitaliano, 1980). The test–retest reliability between the first round and follow-up visit (with 79% of subjects within one quintile of their earlier response) was acceptable for epidemiological purposes, although the kappa value (0.21) was in the zone denoting relatively poor agreement. Self-assessed skin type, for which the kappa value (0.43) indicates moderate reliability, has been shown to correlate well with the gold standard of measured minimal erythema dose (MED) (Weinstock, 1992).

Turning to the substantive results, almost a quarter of subjects had at least one solar keratosis at baseline and one in eight developed at least one new solar keratosis during the follow-up period. One in fifty subjects had an undiagnosed skin cancer (either NMSC or MM) during the prevalence round and just under 1% developed a new NMSC per annum of follow up.

A clearer appreciation of these descriptive findings emerges from comparison with international data (Frost and Green, 1994). In Australia two large studies have indicated a prevalence of SKs in those over 40 years of between 38% (Goodman *et al.*, 1984) and 57% (Marks *et al.*, 1983). From the United States, SK prevalences of 16% (in those over 21 years) (Zagula-Malley *et al.*, 1974) and 10% (65–74 year olds) (Johnson and Roberts, 1978) have been reported. The only previous population-based study from North-West Europe revealed a prevalence of SKs among those over 60 years of 24% (O'Beirn *et al.*, 1968), but had wide confidence limits.

Reported prevalence of NMSC has ranged from 2–3% (Goodman *et al.*, 1984; Marks *et al.*, 1983) (over 40 years) in Australia to 3.6% (65–74 years) (Johnson and Roberts, 1978) in the USA and a histologically verified prevalence of 2% (O'Beirn *et al.*, 1968) (over 60 years) in the west of Ireland, again with wide confidence limits. Age-specific incidence rates for NMSC from an Australian population-based study have been 23/1000 per year (60–69-year-olds) and 50/1000 per year (70-year-olds and above) (Marks *et al.*, 1989).

It is clear from these comparisons that, although generally less common than in sunnier climates like that of Australia and the southern USA, the prevalence and incidence of SKs and NMSCs in this UK random population sample is at a level denoting major clinical and public health significance. In terms of the relative emphases placed upon NMSC and MM it is, moreover, likely that health care expenditure on NMSC is actually greater (Maize, 1986). Interest should not therefore focus upon malignant melanoma to the detriment of NMSC, as has tended to occur in this country.

The natural history of solar keratoses is of particular importance because of the considerable resources devoted worldwide to their obliteration. As with other pre-malignant lesions, such as those of the cervix, our understanding of their natural history is relatively poor. Spontaneous remission of SKs has only been quantified in one other longitudinal study (Marks *et al*., 1986), in Australia, in which 10% of SKs regressed over a one year period, compared with an annualised proportion of 15% in this study. The same Australian study has also estimated the rate of malignant transformation of SKs as 0.75/1000 SK per year (Marks *et al*., 1988), again consistent with the rate of 0/1000 SK per year (95% CI 0-11) found in this study. The combination of a high spontaneous regression and low malignant transformation rate in studies in two countries raises a major question mark over the wisdom of assiduously treating all detected SKs (Marks, 1991).

Finally, this study presents evidence concerning the sun and ultraviolet exposure behaviour of this elderly population. The findings invite comparison with those of the 1993 and 1994 Omnibus Surveys undertaken in the UK by the Office of Population Censuses and Surveys (OPCS). Reported use of sunbeds/UV lamps was significantly higher in this study than that reported in the 1994 survey for the age group 55 years and over (1.1%) (Anonymous, 1994). This may indicate a recent decline in sunbed use as a result of increasing knowledge of their potential hazards, but could equally reflect geographical variation and/or differences in ascertainment. The period prevalence of sunburn in this study is similar to that found in the 1993 OPCS Omnibus survey for this age group (Melia and Bulman, 1995).

Other findings, principally the analytical epidemiological results and an evaluation of preventive measures including sunscreen use, will be presented in a companion paper (Harvey *et al*., 1996). These descriptive findings provide important baseline data, comparison with which will allow, in due course, an overall evaluation of the current programme of primary skin cancer prevention in the UK.

**Acknowledgements**
We wish to acknowledge the financial support for this project from the Welsh Scheme for Health and Social Research. We also thank Dr Tim Peters, Senior Lecturer in Medical Statistics in the University of Bristol, for his helpful advice.

### References

ALTMAN DG. (1991). *Practical Statistics for Medical Research*. pp. 404-408. Chapman and Hall: London.

ANONYMOUS. (1992). *The Health of the Nation: a Strategy for Health in England*. HMSO: London.

ANONYMOUS. (1993). *The Health of the Nation Key Area Handbook: Cancers*. Department of Health: London.

ANONYMOUS. (1994). *OPCS Omnibus Survey*. Office of Populations, Censuses and Surveys: London.

ARMSTRONG BK. (1994). Stratospheric ozone and health. *Int. J. Epidemiol*., **23**, 873-885.

BEADLE PC, BULLOCK D, BEDFORD G, LEACH JF, WEBB RA, DENT NA AND BURTON JL. (1982). Accuracy of skin cancer incidence data in the United Kingdom. *Clin. Exp. Dermatol*., **7**, 255-260.

BICKLER G AND SUTTON S. (1993). Inaccuracy of FHSA registers: help from electoral registers. *Br. Med. J*., **306**, 116.

BONETT A, RODER D AND ESTERMAN A. (1989). Epidemiological features of melanoma in South Australia: implications for cancer control. *Med. J. Aust*., **151**, 502-509.

BRUHL C AND CRUTZEN PJ. (1989). On the disproportionate role of tropospheric ozone as a filter against solar UV-B radiation. *Geophys. Res. Letters*, **16**, 703-706.

CHUANG T-Y, POPESCU A, SU WPD AND CHUTE CG. (1990). Basal cell carcinoma: a population based incidence study in Rochester, Minnesota. *J. Am. Acad. Dermatol*., **22**, 413-417.

DAHLBACK A AND MOAN J. (1990). Annual exposures to carcinogenic radiation from the sun at different latitudes and amplification factors related to ozone depletion. The use of different geometrical representations of the skin surface receiving the ultraviolet radiation. *Photochem. Photobiol*., **52**, 1025-1028.

ELDER DE. (1995). Skin cancer. *Cancer*, **75**, 245-256.

FROST CA AND GREEN AC. (1994). Epidemiology of solar keratoses. *Br. J. Dermatol*., **131**, 455-464.

GAFA L, FILIPPAZZO MG, TUMINO R, DARDANONI G, LANZARONE F AND DARDANONI L. (1991). Risk factors of nonmelanoma skin cancer in Ragusa, Sicily: a case-control study. *Cancer Causes Control*, **2**, 395-399.

GALLAGHER RP, MA B, MCLEAN DI, YANG CP, HO V, CARRUTHERS JA AND WARSHAWSKI LM. (1990). Trends in basal cell carcinoma, squamous cell carcinoma, and melanoma of the skin from 1973 through 1987. *J. Am. Acad. Dermatol*., **23**, 413-421.

GILES GG, MARKS R AND FOLEY P. (1988). Incidence of non-melanocytic skin cancer treated in Australia. *Br. Med. J*., **296**, 13-17.

GOODMAN GJ, MARKS R, SELWOOD TS, PONSFORD MW AND PAKES W. (1984). Non-melanotic skin cancer and solar keratoses in Victoria - clinical studies II. *Aust. J. Dermatol*., **25**, 103-106.

GRAHAM S, MARSHALL J, HAUGHEY B, STOLL H, ZIELEZNY M, BRASURE J AND WEST D. (1985). An inquiry into the epidemiology of melanoma. *Am. J. Epidemiol*., **122**, 606-619.

GREEN A, LESLIE D AND WEEDON D. (1988). Diagnosis of skin cancer in the general population: clinical accuracy in the Nambour survey. *Med. J. Aust*., **148**, 447-450.

HARVEY IM, SHALOM D, MARKS R AND FRANKEL SJ. (1989). Non-melanoma skin cancer. *Br. Med. J*., **299**, 1118-1120.

HARVEY IM, FRANKEL SJ, MARKS R, SHALOM D AND NOLAN-FARRELL M. (1996). Non melanoma skin cancer and solar keratoses in the UK. II. Analytical results of the South Wales skin cancer study. *Br. J. Cancer*, (in press).

JOHNSON M-LT AND ROBERTS J. (1978). Skin conditions and related need for medical care among persons 1-74 years. US Dept of Health, Education and Welfare: Washington, DC.

KROL S, KEIJSER LMT, VAN DER RHEE HJ AND WELVAART K. (1991). Screening for skin cancer in the Netherlands. *Acta Derm. Venereol. (Stockh.)*, **71**, 317-321.

LANE-BROWN MM, SHARPE CAB, MACMILLAN DS AND MCGOVERN VJ. (1971). Genetic predisposition to melanoma and other skin cancers in Australians. *Med. J. Aust*., **1**, 852-853.

MACKIE RM, SOUTAR DS AND WATSON ACH, MCLAREN KM, MCPHIE JL, HUTCHEON AW, SMYTH JF, CALMAN KC, HUNTER JAA, MACGILLIVRAY JB, RANKIN R AND KEMP IW. (1985). Malignant melanoma in Scotland 1979-1983. *Lancet*, **2**, 859-862.

MAIZE JC. (1986). Can the lessons learned from the study of malignant melanoma be extrapolated to other cutaneous neoplasms. *Am. J. Dermatopathol*., **8**, 93-94.

MARKS R. (1986). Premalignant disease of the epidermis. *J.R. Coll. Phys. Lond*., **20**, 116-121.

MARKS R. (1987). Nonmelanotic skin cancer and solar keratoses: the quiet 20th century epidemic. *Int. J. Dermatol*., **26**, 201-205.

MARKS R. (1990). Prevention of skin cancer: being sunsmart in the 1990s. *J. Dermatol. Treat*., **1**, 271-274.

MARKS R. (1991). The role of treatment of actinic keratoses in the prevention of morbidity and mortality due to squamous cell carcinoma. *Arch. Dermatol*., **127**, 1031-1033.

MARKS R. (1995). An overview of skin cancers: incidence and causation. *Cancer*, **75**, 607-612.

MARKS R, PONSFORD MW, SELWOOD TS, GOODMAN G AND MASON G. (1983). Non-melanotic skin cancer and solar keratoses in Victoria. *Med. J. Aust*., **2**, 619-622.

MARKS R, FOLEY P, GOODMAN G, HAGE BH AND SELWOOD TS. (1986). Spontaneous remission of solar keratoses: the case for conservative management. *Br. J. Dermatol*., **155**, 649-655.

MARKS R, RENNIE G AND SELWOOD TS. (1988). Malignant transformation of solar keratoses to squamous cell carcinoma. *Lancet*, **1**, 795-797.

MARKS R, JOLLEY D, DOREVITCH AP AND SELWOOD T. (1989). The incidence of non-melanocytic skin cancers in Australian population: results of a five-year prospective study. *Med. J. Aust*., **150**, 475-478.

MARKS R, STAPLES M AND GILES GG. (1993). Trends in non-melanocytic skin cancer treated in Australia: the second national survey. *Int. J. Cancer*, **53**, 585–590.

MARSHALL VW. (1987). Factors affecting response and completion rates in some Canadian studies. *Can. J. Aging*, **6**, 217–227.

MELIA J AND BULMAN A. (1995). Sunburn and tanning in a British population. *J. Public Health Med.*, **17**, 223–229.

MELIA J, ELLMAN R AND CHAMBERLAIN J. (1994). Meeting *The Health of the Nation* target for skin cancer: problems with tackling prevention and monitoring trends. *J. Public Health Med.*, **16**, 225–232.

NIXON RL, DOREVITCH AP AND MARKS R. (1986). Squamous cell carcinoma of the skin: accuracy of clinical diagnosis and outcome of follow-up in Australia. *Med. J. Aust.*, **144**, 235–239.

O'BEIRN SFO, JUDGE P, URBACH F, MACCON CF AND MARTIN F. (1968). Skin cancer in County Galway, Ireland. In *Proceedings of the Sixth National Cancer Conference*. pp. 489–500. JB Lippincott Co.: Philadelphia

PONSFORD MW, GOODMAN G AND MARKS R. (1983). The prevalence and accuracy of diagnosis of non-melanotic skin cancer in Victoria. *Aust. J. Dermatol.*, **24**, 79–82.

POPESCU NA, BEARD CM, TREACY PJ, WINKELMAN RK, O'BRIEN PC AND KURLAND LT. (1990). Cutaneous malignant melanoma in Rochester, Minnesota: trends in incidence and survivorship. *Mayo Clin. Proc.*, **65**, 1293–1302.

ROBERTS HR, RUSHTON L, MUIR KR, DENGLER P, COUPLAND CAC, JENKINSON CM, RUFFELL A AND CHILVERS CED. (1995). The use of family health services authority registers as a sampling frame in the UK: a review of theory and practice. *J. Epidemiol. Community Health*, **49**, 344–347.

SCOTTO J AND FEARS TR. (1978). Skin cancer epidemiology: research needs. *Natl Cancer Inst. Monogr.*, **50**, 169–177.

STREETLY A AND MARKOWE H. (1995). Changing trends in the epidemiology of malignant melanoma: gender differences and their implications for public health. *Int. J. Epidemiol.*, **24**, 897–907.

URBACH F AND VITALIANO PP. (1980). The relative importance of risk factors in nonmelanoma carcinoma. *Arch. Dermatol.*, **116**, 454–456.

VITASA BC, TAYLOR HR, STRICKLAND PT, ROSENTHAL FS, WEST S, ABBEY H, MUNOZ B AND EMMETT EA. (1990). Association of nonmelanoma skin cancer and actinic keratosis with cumulative solar ultraviolet exposure in Maryland watermen. *Cancer*, **65**, 2811–2817.

WEINSTOCK MA. (1992). Assessment of sun sensitivity by questionnaire: validity of items and formulation of a prediction rule. *J. Clin. Epidemiol.*, **45**, 547–552.

ZAGULA-MALLY ZW, ROSENBERG WE AND KASHGARIAN M. (1974). Frequency of skin cancer and solar keratoses in a rural southern county as determined by population sampling. *Cancer*, **34**, 345–349.


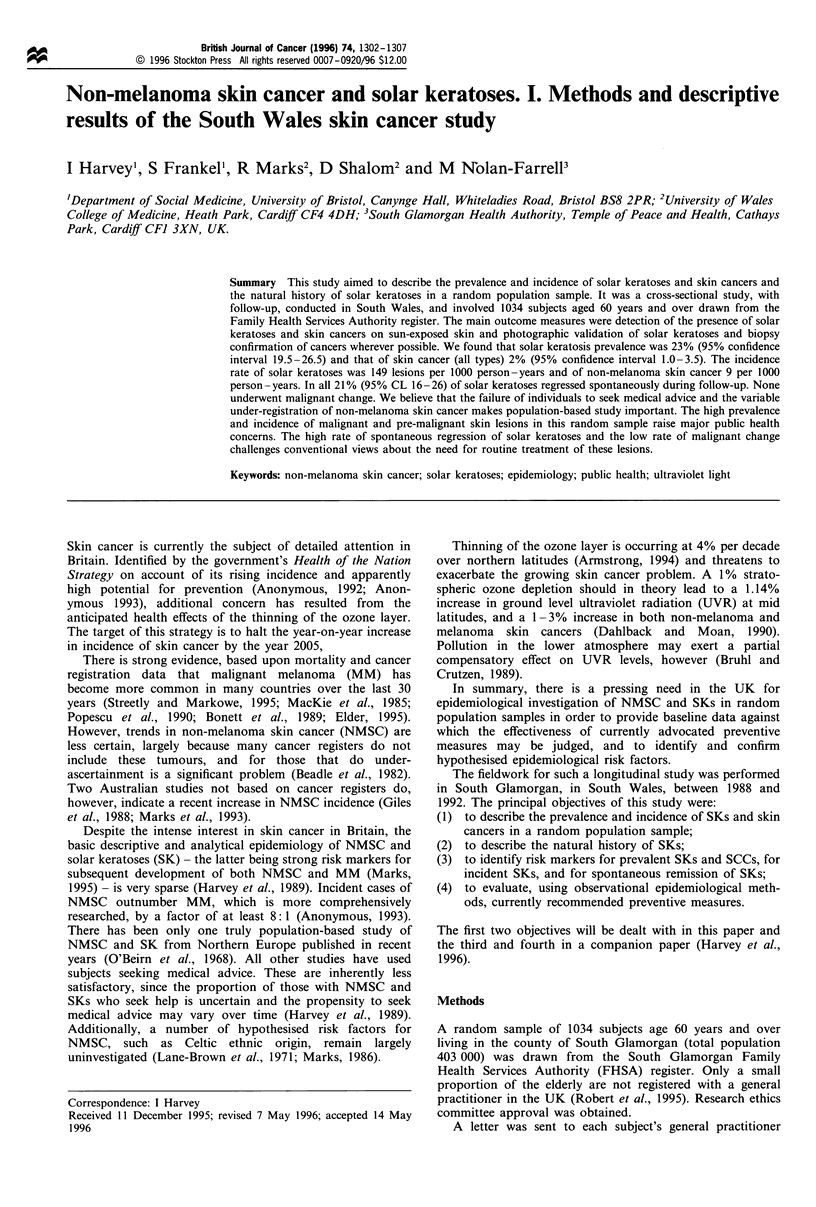

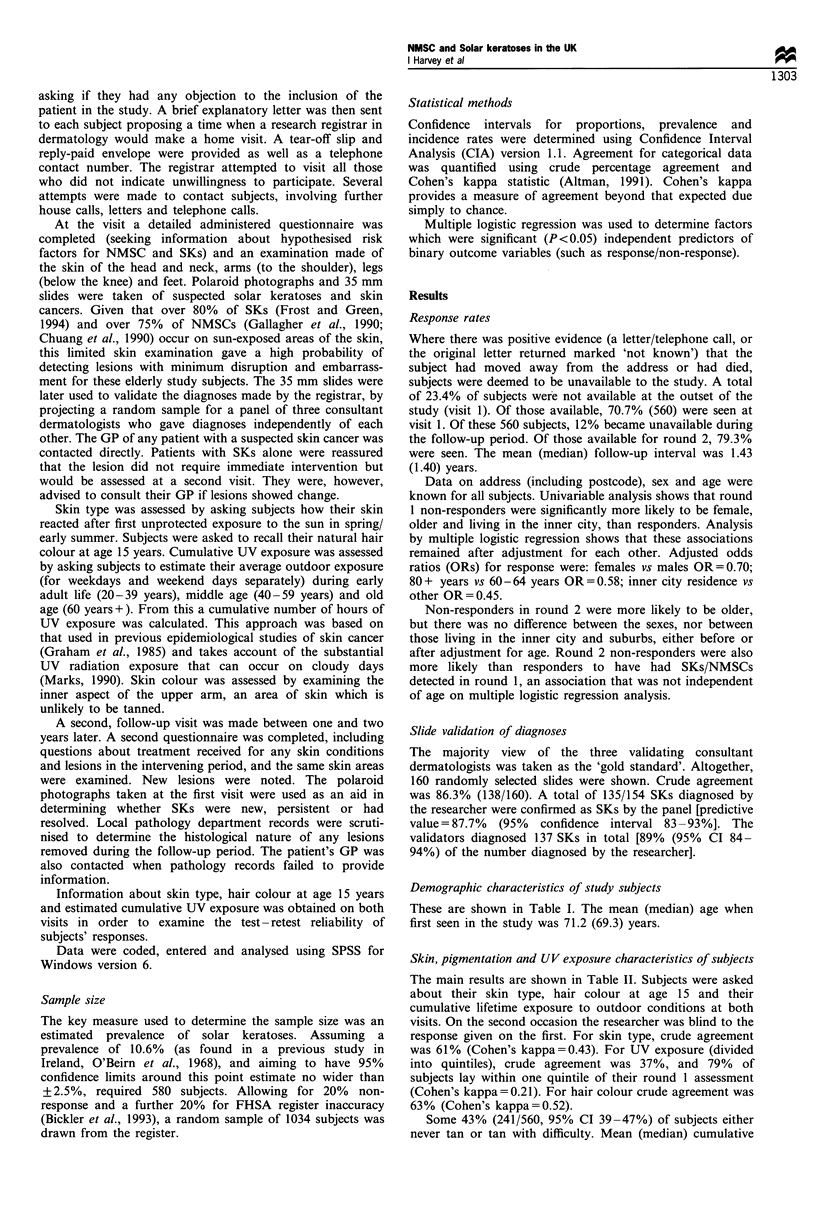

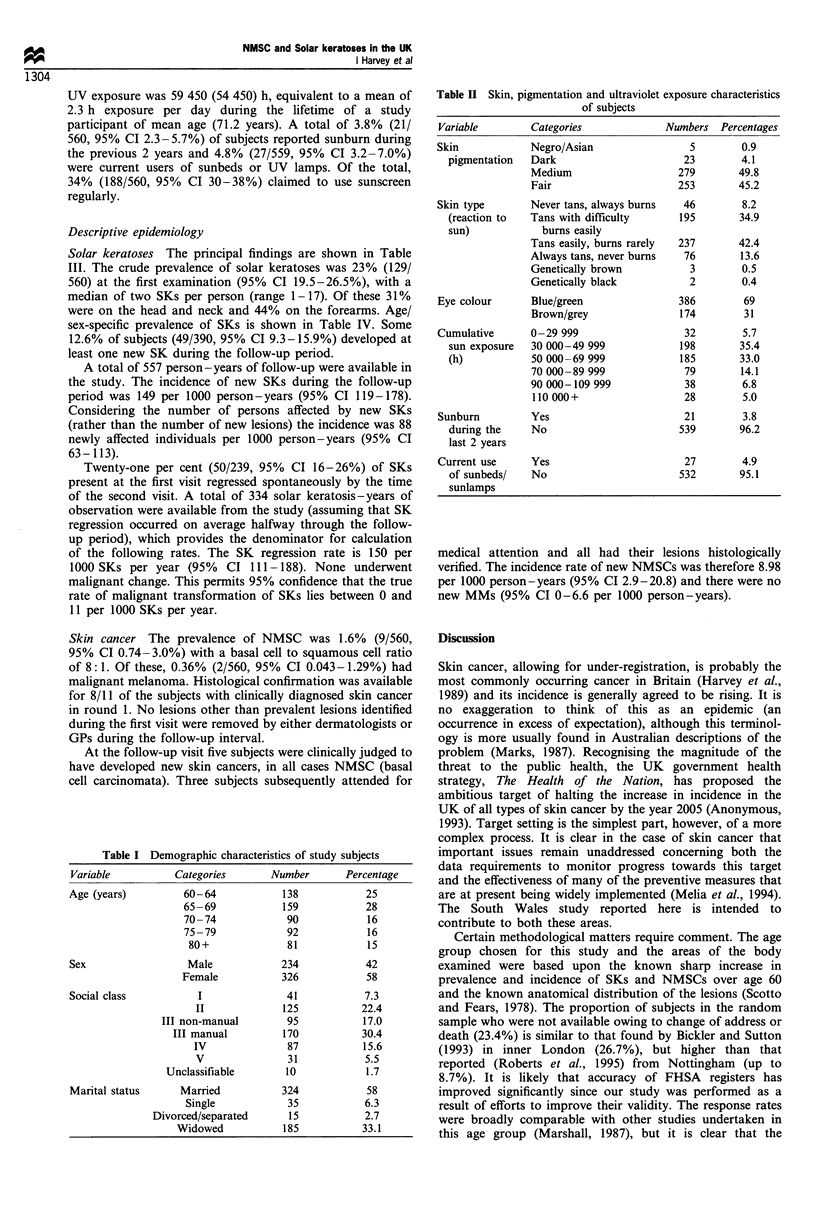

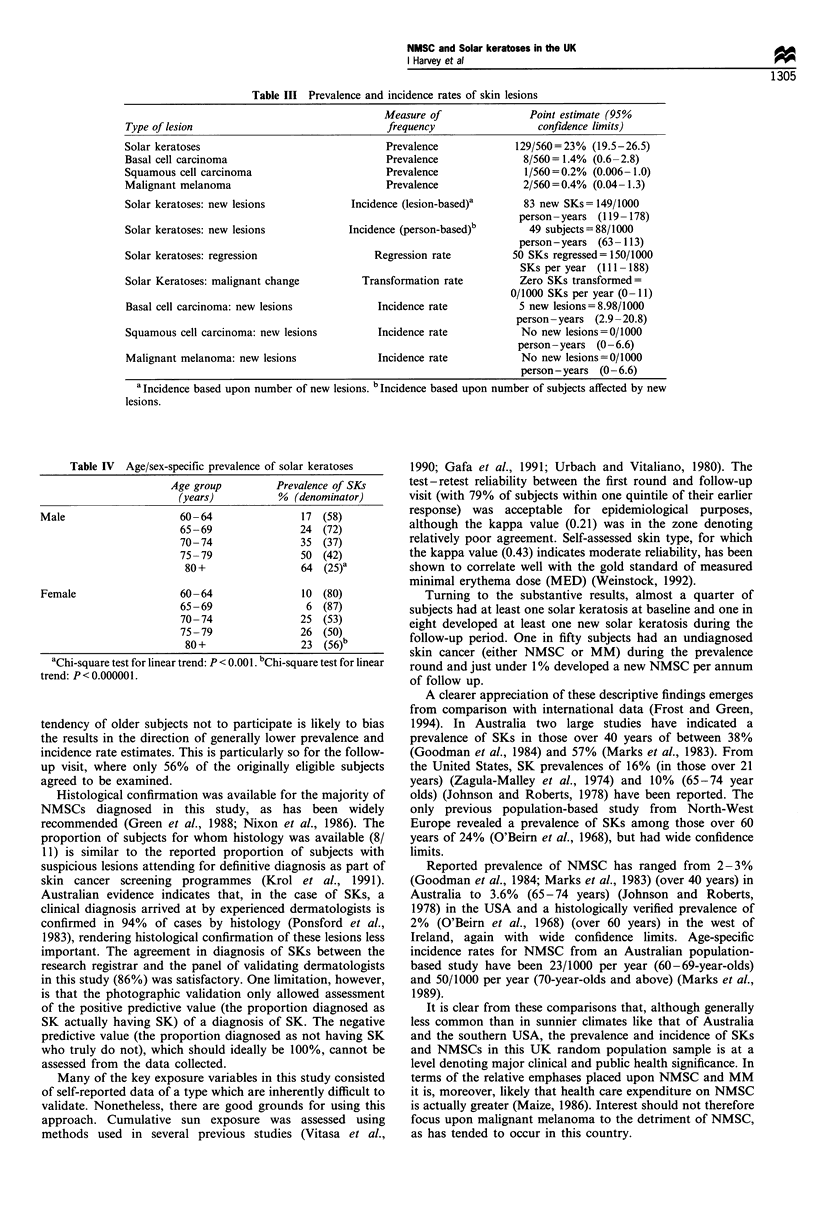

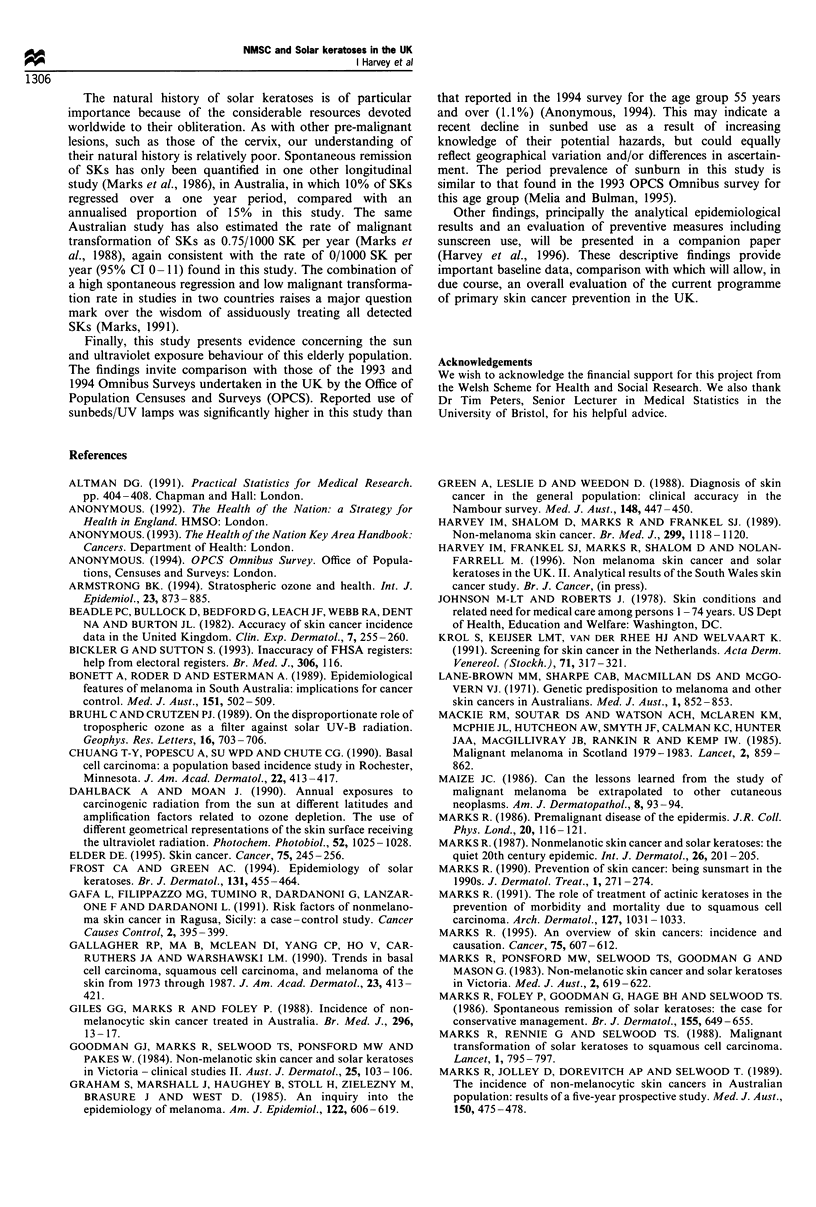

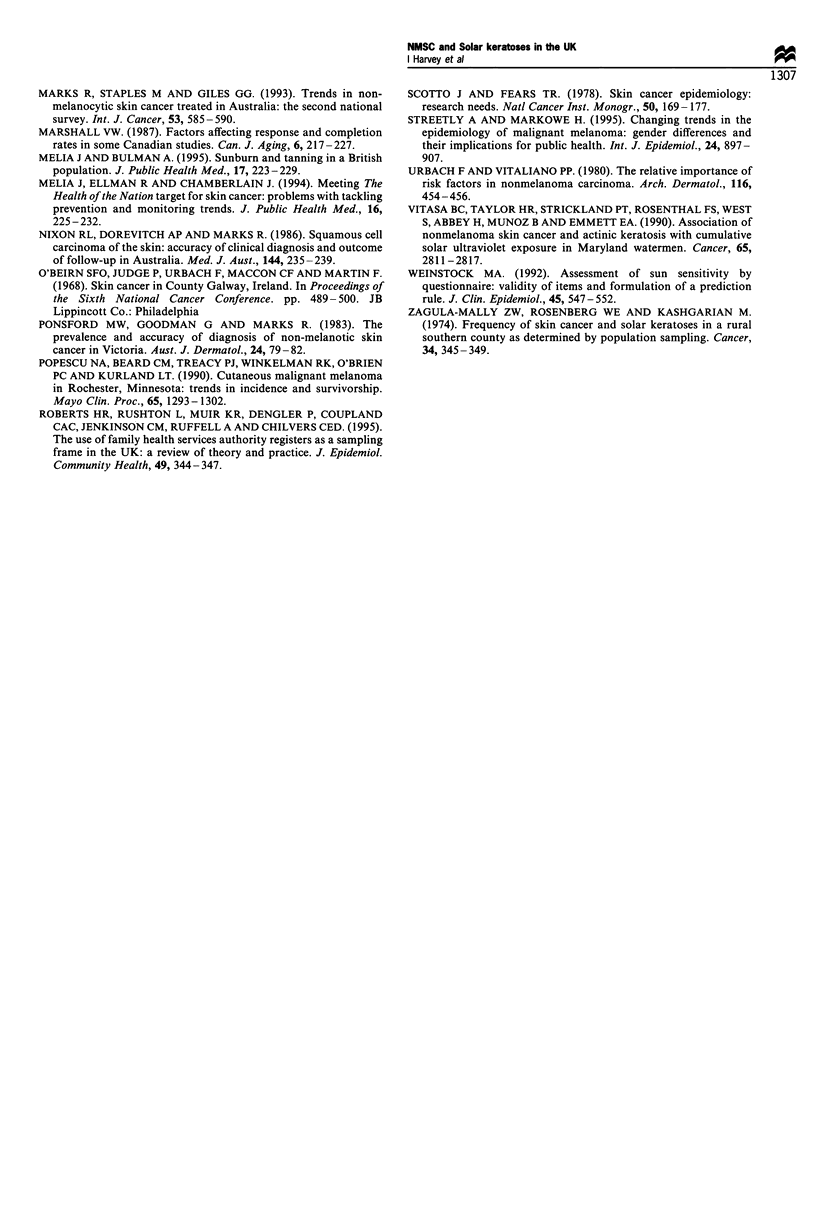


## References

[OCR_00762] Armstrong B. K. (1994). Stratospheric ozone and health.. Int J Epidemiol.

[OCR_00764] Beadle P. C., Bullock D., Bedford G., Leach J. F., Webb R. A., Dent N. A., Burton J. L. (1982). Accuracy of skin cancer incidence data in the United Kingdom.. Clin Exp Dermatol.

[OCR_00775] Bonett A., Roder D., Esterman A. (1989). Epidemiological features of melanoma in South Australia: implications for cancer control.. Med J Aust.

[OCR_00855] Brown M. M., Sharpe C. A., Macmillan D. S., McGovern V. J. (1971). Genetic predisposition to melanoma and other skin cancers in Australians.. Med J Aust.

[OCR_00785] Chuang T. Y., Popescu A., Su W. P., Chute C. G. (1990). Basal cell carcinoma. A population-based incidence study in Rochester, Minnesota.. J Am Acad Dermatol.

[OCR_00790] Dahlback A., Moan J. (1990). Annual exposures to carcinogenic radiation from the sun at different latitudes and amplification factors related to ozone depletion. The use of different geometrical representations of the skin surface receiving the ultraviolet radiation.. Photochem Photobiol.

[OCR_00794] Elder D. E. (1995). Skin cancer. Melanoma and other specific nonmelanoma skin cancers.. Cancer.

[OCR_00798] Frost C. A., Green A. C. (1994). Epidemiology of solar keratoses.. Br J Dermatol.

[OCR_00802] Gafà L., Filippazzo M. G., Tumino R., Dardanoni G., Lanzarone F., Dardanoni L. (1991). Risk factors of nonmelanoma skin cancer in Ragusa, Sicily: a case-control study.. Cancer Causes Control.

[OCR_00808] Gallagher R. P., Ma B., McLean D. I., Yang C. P., Ho V., Carruthers J. A., Warshawski L. M. (1990). Trends in basal cell carcinoma, squamous cell carcinoma, and melanoma of the skin from 1973 through 1987.. J Am Acad Dermatol.

[OCR_00813] Giles G. G., Marks R., Foley P. (1988). Incidence of non-melanocytic skin cancer treated in Australia.. Br Med J (Clin Res Ed).

[OCR_00818] Goodman G. J., Marks R., Selwood T. S., Ponsford M. W., Pakes W. (1984). Non-melanotic skin cancer and solar keratoses in Victoria--clinical studies II.. Australas J Dermatol.

[OCR_00822] Graham S., Marshall J., Haughey B., Stoll H., Zielezny M., Brasure J., West D. (1985). An inquiry into the epidemiology of melanoma.. Am J Epidemiol.

[OCR_00829] Green A., Leslie D., Weedon D. (1988). Diagnosis of skin cancer in the general population: clinical accuracy in the Nambour survey.. Med J Aust.

[OCR_00834] Harvey I., Shalom D., Marks R. M., Frankel S. J. (1989). Non-melanoma skin cancer.. BMJ.

[OCR_00849] Krol S., Keijser L. M., van der Rhee H. J., Welvaart K. (1991). Screening for skin cancer in The Netherlands.. Acta Derm Venereol.

[OCR_00857] MacKie R. M., Smyth J. F., Soutar D. S., Calman K. C., Watson A. C., Hunter J. A., McLaren K. M., MacGillivray J. B., McPhie J. L., Rankin R. (1985). Malignant melanoma in Scotland 1979-1983.. Lancet.

[OCR_00866] Maize J. C. (1986). Can the lesions learned from the study of malignant melanoma be extrapolated to other cutaneous neoplasms?. Am J Dermatopathol.

[OCR_00886] Marks R. (1995). An overview of skin cancers. Incidence and causation.. Cancer.

[OCR_00895] Marks R., Foley P., Goodman G., Hage B. H., Selwood T. S. (1986). Spontaneous remission of solar keratoses: the case for conservative management.. Br J Dermatol.

[OCR_00905] Marks R., Jolley D., Dorevitch A. P., Selwood T. S. (1989). The incidence of non-melanocytic skin cancers in an Australian population: results of a five-year prospective study.. Med J Aust.

[OCR_00875] Marks R. (1987). Nonmelanotic skin cancer and solar keratoses. The quiet 20th century epidemic.. Int J Dermatol.

[OCR_00893] Marks R., Ponsford M. W., Selwood T. S., Goodman G., Mason G. (1983). Non-melanotic skin cancer and solar keratoses in Victoria.. Med J Aust.

[OCR_00871] Marks R. (1986). Premalignant disease of the epidermis. The Parkes Weber lecture 1985.. J R Coll Physicians Lond.

[OCR_00900] Marks R., Rennie G., Selwood T. S. (1988). Malignant transformation of solar keratoses to squamous cell carcinoma.. Lancet.

[OCR_00918] Marks R., Staples M., Giles G. G. (1993). Trends in non-melanocytic skin cancer treated in Australia: the second national survey.. Int J Cancer.

[OCR_00881] Marks R. (1991). The role of treatment of actinic keratoses in the prevention of morbidity and mortality due to squamous cell carcinoma.. Arch Dermatol.

[OCR_00927] Melia J., Bulman A. (1995). Sunburn and tanning in a British population.. J Public Health Med.

[OCR_00931] Melia J., Ellman R., Chamberlain J. (1994). Meeting The Health of the Nation target for skin cancer: problems with tackling prevention and monitoring trends.. J Public Health Med.

[OCR_00935] Nixon R. L., Dorevitch A. P., Marks R. (1986). Squamous cell carcinoma of the skin. Accuracy of clinical diagnosis and outcome of follow-up in Australia.. Med J Aust.

[OCR_00948] Ponsford M. W., Goodman G., Marks R. (1983). The prevalence and accuracy of diagnosis of non-melanotic skin cancer in Victoria.. Australas J Dermatol.

[OCR_00954] Popescu N. A., Beard C. M., Treacy P. J., Winkelmann R. K., O'Brien P. C., Kurland L. T. (1990). Cutaneous malignant melanoma in Rochester, Minnesota: trends in incidence and survivorship, 1950 through 1985.. Mayo Clin Proc.

[OCR_00960] Roberts H. R., Rushton L., Muir K. R., Dengler R., Coupland C. A., Jenkinson C. M., Ruffell A., Chilvers C. E. (1995). The use of family health services authority registers as a sampling frame in the UK: a review of theory and practice.. J Epidemiol Community Health.

[OCR_00966] Scotto J., Fears T. R. (1978). Skin cancer epidemiology: research needs.. Natl Cancer Inst Monogr.

[OCR_00970] Streetly A., Markowe H. (1995). Changing trends in the epidemiology of malignant melanoma: gender differences and their implications for public health.. Int J Epidemiol.

[OCR_00976] Vitaliano P. P., Urbach F. (1980). The relative importance of risk factors in nonmelanoma carcinoma.. Arch Dermatol.

[OCR_00981] Vitasa B. C., Taylor H. R., Strickland P. T., Rosenthal F. S., West S., Abbey H., Ng S. K., Munoz B., Emmett E. A. (1990). Association of nonmelanoma skin cancer and actinic keratosis with cumulative solar ultraviolet exposure in Maryland watermen.. Cancer.

[OCR_00986] Weinstock M. A. (1992). Assessment of sun sensitivity by questionnaire: validity of items and formulation of a prediction rule.. J Clin Epidemiol.

[OCR_00991] Zagula-Mally Z. W., Rosenberg E. W., Kashgarian M. (1974). Frequency of skin cancer and solar keratoses in a rural southern county as determined by population sampling.. Cancer.

